# Mood changes of children and adolescents in dance classes: a prospective repeated-measures study

**DOI:** 10.3389/fpsyg.2026.1719704

**Published:** 2026-06-11

**Authors:** Tiffany C. Henderson, Paul R. Henderson, Claire Robertson-Kraft, Judy Saltzberg Levick

**Affiliations:** 1 Master of Applied Positive Psychology Program, University of Pennsylvania, Philadelphia, PA, United States; 2 Tiffany’s Dance Academy, Livermore, CA, United States; 3 Raising the Barre, Livermore, CA, United States

**Keywords:** children and adolescents, dance education, ecological momentary assessment, mood, multilevel modeling, PANAS-C, positive psychology, well-being

## Abstract

**Introduction:**

Adolescent mental health in the United States has deteriorated markedly over the past decade, with diagnosed mental or behavioral health conditions among 12-to-17-year-olds increasing 35% between 2016 and 2023, driven by a 61% rise in anxiety diagnoses and a 45% rise in depression. Community-based interventions combining physical activity, creative expression, and social connection represent a promising response.

**Methods:**

This prospective repeated-measures study examined the association between structured dance education and mood among 256 children and adolescents (ages 5–17) at four studio locations. Over 4 months (February–May 2025), students completed 4,224 paired pre- and post-class mood ratings using a single-item emoji scale; the primary analytic sample comprised 4,059 sessions from 251 students. PANAS-C administrations provided a concurrent measure of dispositional affect, with each student’s score anchored to the administration nearest the study close. Multilevel models accounted for the nested data structure (students contributing 1–109 sessions each).

**Results:**

The intraclass correlation was 0.432. Post-class mood was significantly higher than pre-class mood (session-level Cohen’s d = 0.27 [primary estimate] and per-student d_z = 0.43 [*p* < 0.001]) with 85.8% of sessions showing maintained or improved mood. A Tobit sensitivity analysis confirmed these patterns after adjusting for the 28.8% post-class ceiling rate. No covariate (including weekly frequency, class type, proficiency level, or instructor experience) explained significant additional variance. Individual differences dominated: random slopes improved model fit substantially (ΔAIC = 201). Convergent validity between the emoji scale and PANAS-C Positive Affect was significant (*r* = 0.19, *p* = 0.011, *n* = 175), with the strongest association among students attending 4–6 classes per week (*r* = 0.51, *n* = 25).

**Conclusion:**

Dance participation was associated with a consistent acute mood benefit generalizing across genres, schedules, and experience levels; individual characteristics, rather than program features, accounted for most meaningful variation. The observational design precludes causal attribution.

## 1 Introduction

Adolescent mental health in the United States has deteriorated markedly over the past decade, with diagnosed mental or behavioral health conditions among 12-to-17-year-olds increasing 35% between 2016 and 2023, driven by a 61% rise in anxiety diagnoses and a 45% rise in depression (Daly, 2022; Sappenfield et al., 2024). The 2023 CDC Youth Risk Behavior Survey found that 40% of high school students experienced persistent sadness or hopelessness, rising to 53% among girls (CDC, 2024). These trends coincide with increases in screen time and social media use, both of which are consistently associated with poorer mental health outcomes in adolescents (Twenge et al., 2019; Orben and Przybylski, 2019; Sampasa-Kanyinga et al., 2022). The crisis extends beyond digital harms: a global survey of over 10,000 youth across ten countries found that 59% were “very” or “extremely worried” about climate change, with nearly half reporting that this anxiety affected their daily functioning (Hickman et al., 2021).

Against this backdrop, community-based interventions that promote physical activity, in-person connection, and creative expression are urgently needed. Dance education is a promising candidate. It is among the most widely practiced structured physical activities for youth, with an estimated 6.7 million children and adolescents enrolled in dance programs in the United States alone (National Endowment for the Arts, 2020). Internationally, dance participation rates among young people range from 8 to 25% across Europe, South America, and East Asia (Fong Yan et al., 2018; Tao et al., 2022), though enrollment is disproportionately female and concentrated in middle-to-upper socioeconomic strata.

### 1.1 Dance and psychological well-being

A growing body of evidence links dance participation to improved mood and emotional well-being in young people. Systematic reviews confirm that recreational and structured dance interventions benefit both the physical and psychological health of children and adolescents (Burkhardt and Brennan, 2012; Tao et al., 2022; Fong Yan et al., 2018), with effect sizes comparable to other physical activity interventions (Koch et al., 2014). Studies across diverse settings, including dance-based interventions in the United Kingdom (Moula et al., 2020; Sango and Pickard, 2024), school-based interventions in Australia (Burkhardt and Brennan, 2012), dance therapy programs in Greece (Darginidou et al., 2018), and structured dance curricula in China and South Korea (Tao et al., 2022), consistently report improvements in mood, self-esteem, and emotional expression among youth participants.

What distinguishes dance from conventional physical activity is its simultaneous engagement of multiple psychological pathways. Dance combines physical exertion, which stimulates neurobiological processes including BDNF production and endorphin release (Murawska-Ciałowicz et al., 2021; Ratey, 2013), with creative expression that provides an outlet for emotional processing (Karkou and Sanderson, 2006). It is inherently social: coordinated rhythmic movement fosters interpersonal synchrony, collective joy, and a sense of belonging that reduces isolation (Basso et al., 2021; Ehrenreich, 2007; Phillips-Silver et al., 2010). And it cultivates engagement through flow states that arise from learning choreography and executing complex motor sequences (Bégel et al., 2022; Hackney et al., 2024). This multi-modal quality makes dance more than the sum of its component benefits, it is a single activity that operates across physical, social, emotional, and cognitive domains simultaneously.

### 1.2 Theoretical framing

These mechanisms align with two established theoretical frameworks. Seligman’s (2011) PERMA model identifies five elements of human flourishing, Positive emotions, Engagement, Relationships, Meaning, and Achievement, each of which dance education engages directly. Positive emotions emerge through the joy and uplift of movement to music. Engagement manifests in the flow states required for learning choreography. Relationships are strengthened through group activities and peer interactions. Meaning develops as students connect with cultural traditions and artistic expression. Achievement grows through skill mastery and performance opportunities.

Complementing PERMA, the RAISE framework from the positive humanities (Tay et al., 2018) identifies five psychological mechanisms through which arts engagement promotes flourishing: Reflection (introspection about identity and experience), Acquisition (developing new competencies), Immersion (deep absorption in aesthetic experience), Socialization (building community through shared participation), and Expression (externalizing inner states through creative action). Dance uniquely activates all five mechanisms in each class session: a dancer reflects on their movement quality, acquires technical skills, becomes immersed in music and choreography, socializes through synchronized group work, and expresses emotion through their body. This theoretical convergence suggests that dance may function as a comprehensive positive psychology intervention, addressing multiple pathways to well-being within a single activity.

### 1.3 The present study

Despite growing evidence for dance’s psychological benefits, several gaps remain. Most prior research relies on pre-post designs with small samples and short observation periods, typically examining a single dance program over a few weeks (Burkhardt and Brennan, 2012; Koch et al., 2014). Few studies have tracked mood at the level of individual class sessions across extended time periods, and none, to our knowledge, have used ecological momentary assessment in naturalistic dance studio settings with the kind of repeated-measures density that permits multilevel modeling of within-student mood trajectories. It also remains unclear whether the psychological benefits of dance vary by genre, student experience level, or participation frequency, or whether they generalize broadly across these dimensions.

This study addresses these gaps by examining the immediate and longer-term emotional effects of structured dance education on children and adolescents aged 5–17. Over a four-month period, 256 students across four studio locations completed mood assessments before and after each class session using a custom-built digital tool, yielding 4,224 paired observations. Standardized PANAS-C assessments provided a concurrent measure of dispositional affect. We tested three hypotheses:

*Hypothesis 1 (H1)*: Dance classes will be associated with significant mood improvements from pre-class to post-class measurements, with participants showing higher mood ratings after class compared to before class.

*Hypothesis 2 (H2)*: Students' average post-class mood ratings will show positive convergent validity with their PANAS-C Positive Affect scores, providing evidence that the momentary mood measure captures meaningful affective variance.

*Hypothesis 3 (H3)*: Students taking more dance classes per week will demonstrate stronger psychological benefits compared to those taking fewer classes, consistent with a dose-response pattern between dance frequency and emotional outcomes.

## 2 Method

### 2.1 Research design

This study employed a longitudinal observational design with repeated measures to examine the association between structured dance participation and emotional well-being in children and adolescents. Both immediate mood assessments and longitudinal well-being data were collected over a four-month period from February 19 to May 31, 2025, with follow-up assessments planned through 2026. This design allowed for examination of both immediate associations between dance participation and mood, as well as sustained changes in psychological well-being over time, addressing limitations identified in previous dance research that often relied on single-time point measurements (Karkou and Sanderson, 2006).

In addition to examining raw pre–post mood change, we created categorical mood outcomes that helped us track how students’ feelings shifted during class while accounting for the fact that many already began in a positive state (a ceiling effect). These categories were: *Joy Maintenance* (started high ≥5 and stayed high), *Joy Loss* (started high and dropped ≥1 point), *Joy Attainment* (started below 5 and rose to ≥5), and *Joy Missed* (started below 5 and remained below 5). This approach provided a more nuanced view of mood dynamics than mean scores alone and allowed us to capture both stability and change in affect.

### 2.2 Participants

The study recruited participants from 753 children and adolescents aged 5–17 years enrolled in dance classes at Tiffany’s Dance Academy locations across four California cities: Livermore, Danville, Fremont, and San Ramon. Inclusion criteria required participants to be between the ages of 5 and 17 years, currently enrolled in dance classes at Tiffany’s Dance Academy, maintain regular class attendance, and provide appropriate parental consent and child assent following established ethical guidelines for research with minors (Society for Research in Child Development, 2007). Exclusion criteria eliminated children under age 5, dancers over 18, individuals unable to complete mood tracking assessments, those with irregular class attendance, and those lacking proper consent documentation.

Families were recruited through a multi-channel approach beginning in January 2025. Studio administrators distributed informational flyers at all four locations and sent recruitment emails to families of all enrolled students aged 5–17. A brief verbal announcement was made in classes during the first 2 weeks of the recruitment window. Interested families received a detailed study information packet via email, including a plain-language summary of the study’s purpose, procedures, time commitment, and data privacy protections. Parental consent forms and child assent forms were collected electronically through a secure Salesforce portal before any data collection began. No incentives were offered for participation. Recruitment remained open throughout the study period (February 19–May 31, 2025) to accommodate late enrollees, though the majority of consents were obtained during the first 4 weeks.

The study aimed to recruit 200 participants across different dance genres to ensure adequate statistical power and representation across age groups and dance styles, following power analysis recommendations for repeated measures designs (Cohen, 1988). During the study period from February 19, 2025 to May 31, 2025, Tiffany’s Dance Academy had 753 students enrolled across its four locations. Of these, 534 students were between the ages of 5 and 17 and met the eligibility criteria for research participation. Among eligible students, 261 provided both parental consent and child assent, which means 48.9 percent of the eligible population took part in the study. An additional five students initially consented but withdrew before completing the study, resulting in a final analytic sample of 256 participants. The group of consented participants had a slightly higher average age, at 9.2 years, compared to 8.7 years for non-consented students. Preteens made up a larger share of the consented group, while younger children, especially those ages 5 and 6, were more common among non-consented students. Both groups were predominantly female, which matches the typical gender distribution in youth dance programs. In the consented group, 94 percent identified as female, 5.6 percent as male, and 0.4 percent as other or unspecified. The non-consented group included 96 percent female, 3.7 percent male, and 0.3 percent other or unspecified. Overall, the research sample closely reflects the demographics of the eligible student body, though preteens are slightly overrepresented. These patterns suggest that the study’s findings are most applicable to preteens and older children in dance, while results for younger children should be interpreted with some caution due to lower participation in that age group. The small number of withdrawals (five students) represents less than 2 % of the consented sample and is unlikely to affect the overall representativeness of the study. Race and ethnicity data were not collected as part of this study. The IRB-approved protocol prioritized minimizing participant burden for young children, and the primary research questions concerned within-subject mood change rather than between-group demographic comparisons. This limits the generalizability of findings across racial and ethnic groups and is discussed as a limitation.

The final analytic dataset comprised 256 participants contributing 4,224 paired pre–post mood assessments across Ballet, Jazz, Hip Hop, Pointe, Contemporary, Combo, and Performing Company Rehearsal classes. Excluded class types (e.g., Tap, Lyrical, Other, Twinkle Stars, and Company Technique) were omitted from class-type analyses to preserve interpretability. The seven included genres were selected because they represented the studio’s core curriculum and generated sufficient session volume for stable within-genre estimates. Excluded class types had fewer than 30 paired observations or fewer than five unique students (e.g., Tap contributed only 15 sessions from two students), falling below the minimum threshold for meaningful class-type comparisons in the multilevel framework.

Students were classified into four proficiency levels based on the studio’s existing placement system, which considers years of training, technical skill assessments, and instructor recommendations: Beginner (*n* = 98; typically 0–2 years of formal training), Intermediate (*n* = 98; 2–5 years), Advanced (*n* = 54; 5–8 years), and Expert (*n* = 6; 8 + years, including competitive and pre-professional students). These classifications were determined by studio instructors prior to the study and were not modified for research purposes. The Expert category was retained in descriptive analyses but excluded from proficiency-level inferential comparisons due to its small cell size.

### 2.3 Data collection instruments

**Figure 1:**
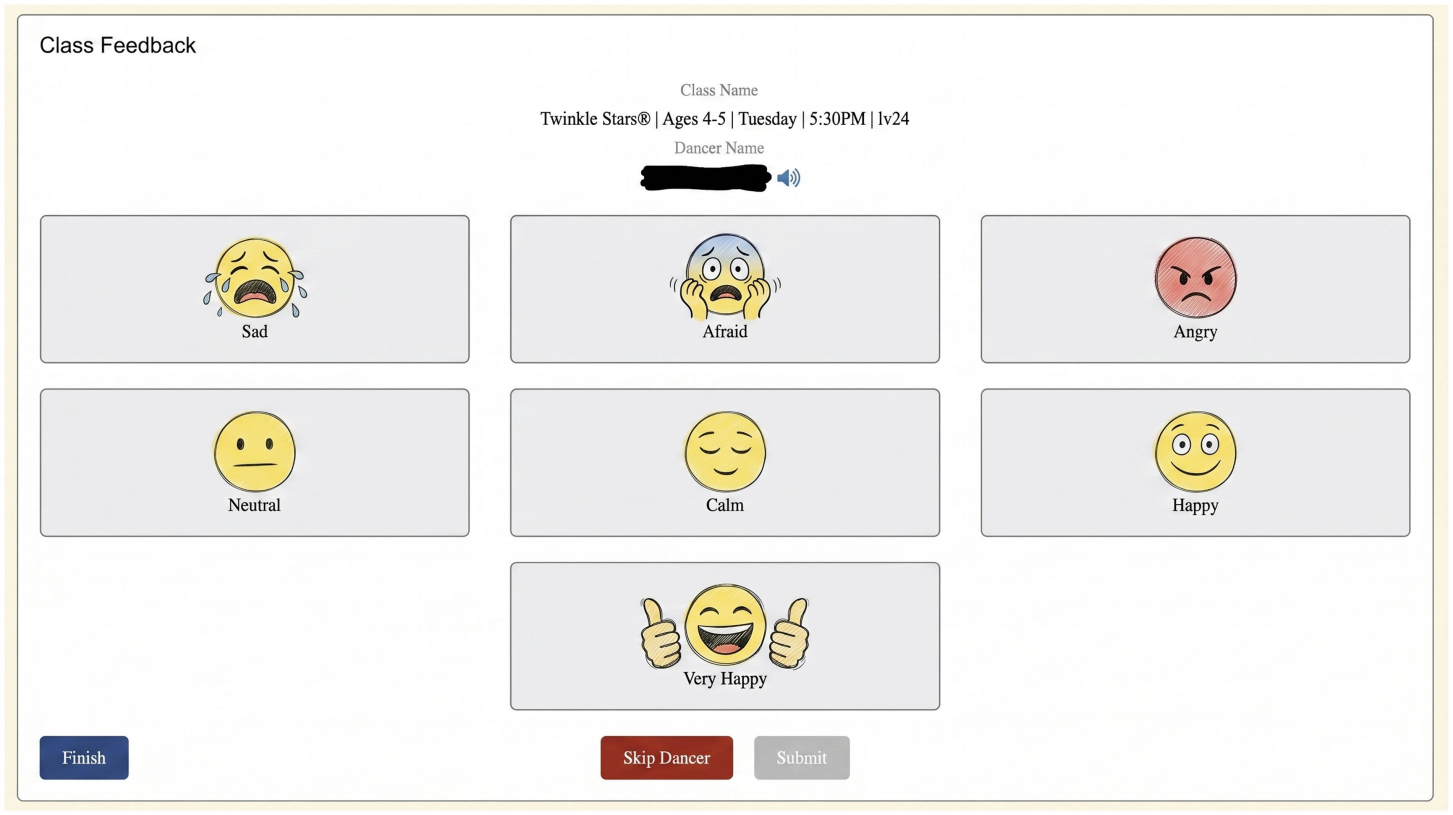
Illustrative recreation of the custom-built tablet application used for immediate mood assessment. Dancers selected one of seven emoji faces corresponding to a single-item mood rating scale (1 = Sad, 2 = Afraid, 3 = Angry, 4 = Neutral, 5 = Calm, 6 = Happy, 7 = Very Happy). Assessments were completed individually before and after each dance class. The application recorded responses with timestamps, student identifiers, and class metadata. Note: Emoji designs shown here are AI-generated stylized renderings (Google Gemini, May 2026) intended for documentation purposes; the deployed application used different emoji designs, but the seven response categories and their ordinal mapping (1 = Sad to 7 = Very Happy) are identical. See Section 2.3 for instrument details and Section 3.6 for convergent validity evidence with PANAS-C positive affect.

Two primary instruments were used for data collection, selected based on established psychometric properties and developmental appropriateness. The immediate mood assessment consisted of a single-item, seven-point mood measure (1 = Sad, 2 = Afraid, 3 = Angry, 4 = Neutral, 5 = Calm, 6 = Happy, 7 = Very Happy.) represented by emoji faces. Students completed mood assessments immediately before and after each dance class using the tablet-based system. Pre-class measurements were collected during the initial 2–3 min as students arrived and checked in, while post-class measurements were administered in the final 2–3 min before dismissal. This timing protocol ensured measurements captured students’ emotional states proximal to the dance experience while minimizing external influences as shown in Figure 1.

Raw pre- and post-class mood scores were transformed into four categorical outcomes based on directional change patterns: (1) Joy Maintenance - students who began class with positive mood (≥5 on the 7-point scale) and maintained or improved their positive state post-class; (2) Attainment - students who began with neutral or negative mood (<5) and achieved positive mood (≥5) post-class; (3) Loss - students who began with positive mood (≥5) but declined to neutral or negative mood (<5) post-class; and (4) Missed - students who began with neutral or negative mood (<5) and remained in that state post-class. This categorical framework allowed analysis of both mood improvement trajectories and the maintenance of positive emotional states, providing insight into dance’s capacity to both elevate and sustain positive affect across different baseline conditions.

This approach follows recommendations for brief, developmentally appropriate mood assessments in educational settings (Dimitrov, 2012) and has demonstrated validity in previous research with youth populations. The validated PANAS-C questionnaire (Laurent et al., 1999) was administered to assess positive and negative affect at regular intervals including baseline assessment prior to first dance class, every 3 months during the dance season, at the end of the dance season (June 2025). The PANAS-C demonstrates strong psychometric properties with reliability coefficients of *α* = 0.90 for positive affect and α = 0.94 for negative affect in youth samples (Laurent et al., 1999).

### 2.4 Data collection procedures

The custom mood-tracking application implemented comprehensive safeguards to ensure data integrity and participant privacy. Individual voice prompts called each dancer’s name while iPad screens were positioned away from instructors and other students to ensure privacy. Participants made individual mood selections with no vocalization of responses, and a three-second delay between participants maintained confidentiality. Time-restricted access limited data collection to scheduled class times plus 10-min grace periods, preventing unauthorized mood entries outside legitimate class sessions and ensuring temporal validity of measurements.

PANAS-C surveys were administered online through a secure Salesforce backend system with automated email generation to parent email addresses containing unique, secure survey links for each participant. Parental assistance was available for younger children while independent completion was encouraged for capable participants, following developmental guidelines for survey administration (Bell, 2007). All data transmission and storage used secure protocols with a unique identifier linking to maintain longitudinal tracking while preserving anonymity, adhering to federal guidelines for educational data protection (Family Educational Rights and Privacy Act, 1974).

Derived metrics (Joy Maintenance, Loss, Attainment, Missed) were computed algorithmically from the mood-tracking app data, while standardized within-subject change was quantified using Cohen’s *d* to provide effect sizes interpretable across educational interventions (Cohen, 1988).

### 2.5 Ethical considerations

This study was approved by the University of Pennsylvania Institutional Review Board and classified as minimal risk. All procedures complied with the ethical standards of APA (2017) and the Declaration of Helsinki. Written informed parental consent and child assent were obtained for all participants prior to data collection; assent procedures were adapted for developmental level, with simplified language and visual aids for children under age 8 (Society for Research in Child Development, 2007). To mitigate potential coercion arising from the dual role of the studio owners as investigators, several safeguards were implemented: recruitment materials explicitly stated that participation was voluntary and would not affect class enrollment or standing; consent was collected by administrative staff rather than studio owners or instructors; mood data were collected via individual tablet stations positioned away from instructors, with no real-time visibility into individual responses; and all data were de-identified before analysis. A detailed conflict of interest disclosure, including financial interests and mitigation strategies, is provided in the Conflict of Interest statement.

Two of the authors (TH and PH) are co-owners of the studio system from which participants were recruited, creating a dual practitioner-researcher role that warrants explicit reflection. This position afforded ecological advantages, including naturalistic access to a large longitudinal sample, familiarity with the instructional context, and the ability to embed data collection seamlessly into class routines, but also introduced interpretive risks. Specifically, the authors’ long-term professional investment in dance education could predispose them toward favorable interpretation of findings. To mitigate this risk, several a priori safeguards were built into the study infrastructure. The mood-tracking application automated the survey process: after an instructor initiated the class session, the application sequentially called each enrolled student by name via voice prompt, and students made individual emoji selections on a tablet positioned away from the instructor and peers (see Section 2.4). Instructors were not present at the tablet during student responses and had no visibility into individual selections. TH, who taught approximately five classes per week, recused herself from initiating surveys in her own classes; a designated assistant performed this step. The application enforced time-restricted data entry, accepting mood submissions only during scheduled class times plus a 10-min grace period, preventing retroactive entry. All analyses followed a pre-specified sequential model-building strategy, and the full analytic pipeline, from data scoring through multilevel modeling, Tobit sensitivity analysis, and figure generation, was constructed to be fully reproducible from de-identified inputs and is publicly available (see Data Availability Statement), enabling independent verification of all reported statistics. Critically, the results reported here include substantive null findings, frequency of participation did not predict post-class mood in multilevel models, no covariate reached significance in the full model, and convergent validity between the emoji scale and PANAS-C was modest (*r* = 0.19), none of which were suppressed or minimized. The transparent reporting of results that did not support the original hypotheses (particularly H3) provides evidence that the conflict mitigation strategies functioned as intended. Readers should nonetheless interpret findings in the context of the investigators’ dual role, and independent replication in settings without practitioner-researcher overlap is encouraged.

### 2.6 Statistical analysis plan

#### 2.6.1 Primary analysis: multilevel models

Because students contributed multiple class sessions (*M* = 16.5, *Mdn* = 9, range = 1–109), observations were not independent. We therefore used multilevel linear models with sessions (Level 1) nested within students (Level 2) as the primary analytic framework, estimated via restricted maximum likelihood using lme4 (Bates et al., 2015) with Satterthwaite degrees of freedom (lmerTest; Kuznetsova et al., 2017).

We followed a sequential model-building strategy. Model 0 (null) estimated the intraclass correlation coefficient (ICC) to quantify the proportion of variance in post-class mood attributable to stable between-student differences. Model 1 added grand-mean-centered pre-class mood as a Level 1 covariate. Model 2 added weekly frequency cohort (1–3, 4–6, 7 + classes per week) as a Level 2 predictor to test for frequency-related differences in post-class mood. Model 3 added the full set of covariates: student proficiency level, class type (Ballet, Jazz, Hip Hop, Contemporary/Lyrical, Pointe, Combo, Company Rehearsal), time of day, day of week, and instructor experience level. Model 4 extended Model 3 by allowing the slope of pre-class mood to vary randomly across students, testing whether the within-session mood relationship differed across individuals. Model 5 tested a crossed random-effects structure by adding instructor as a second grouping factor.

Models were compared using likelihood ratio tests, AIC, and BIC. We report marginal *R*^2^ (variance explained by fixed effects) and conditional *R*^2^ (variance explained by both fixed and random effects) following Nakagawa and Schielzeth (2013).

#### 2.6.2 Sensitivity analysis: ceiling effects

Preliminary inspection revealed that 28.8% of post-class mood ratings reached the scale maximum (7 on a 1–7 scale), indicating substantial right censoring. To assess whether ceiling compression attenuated estimated effects, we fit Tobit regression models (Tobin, 1958) with the upper bound set at 7, using the AER package (Kleiber and Zeileis, 2008). Because Tobit models do not accommodate nested random effects, we computed cluster-robust standard errors at the student level using sandwich estimators (Zeileis, 2004) to account for the within-student correlation structure.

Tobit models paralleled the MLM specification: Model T1 included pre-class mood and frequency cohort; Model T2 added the full covariate set; Model T3 tested a frequency × pre-class mood interaction to examine whether ceiling compression differentially affected frequency cohorts.

#### 2.6.3 Descriptive and exploratory analyses

Session-level paired *t*-tests quantified the overall pre-to-post mood change with Cohen’s *d* as an effect size (Cohen, 1988). A parallel per-student analysis averaged each student’s sessions before testing, yielding Cohen’s *d*_z_. These analyses provide descriptive context and continuity with prior dance-mood research but are not the basis for inferential claims about covariates or moderators.

To characterize mood trajectories beyond mean change, we classified each session into one of four mutually exclusive categories based on a threshold of 5 on the 7-point scale: Joy Maintenance (entered ≥ 5 and remained ≥ 5), Joy Loss (entered ≥ 5 and declined below 5), Joy Attainment (entered < 5 and rose to ≥ 5), and Joy Missed (entered < 5 and remained < 5). These categories capture mood stability and directional shifts that mean change scores can obscure, particularly under ceiling conditions. Proportions are reported descriptively by frequency cohort and class type.

Convergent validity was assessed by correlating each student’s mean post-class mood rating with their PANAS-C Positive Affect score (Laurent et al., 1999), computed at the student level to avoid inflating sample size through repeated sessions. For students who completed the PANAS-C on multiple occasions, the response closest in time to the student’s median mood-assessment date was selected to minimize measurement lag between state and trait assessments. Post-class mood ratings were averaged within a ± 14-day window of each student’s selected PANAS-C response. We report overall and frequency-stratified Pearson correlations with Fisher *z*-transformed 95% confidence intervals.

#### 2.6.4 Software

All analyses were conducted in R 4.5 using lme4 and lmerTest for multilevel models, AER and sandwich for Tobit models with cluster-robust standard errors, performance and MuMIn for model diagnostics, and ggplot2 for figures. The full analysis pipeline, including data preparation, scoring, validation, and modeling scripts, is publicly available at https://github.com/phenlab/dance-mood-replication, along with deidentified analytic data and package version specifications (renv.lock) to support full reproducibility.

## 3 Results

### 3.1 Descriptive overview

**Figure 2:**
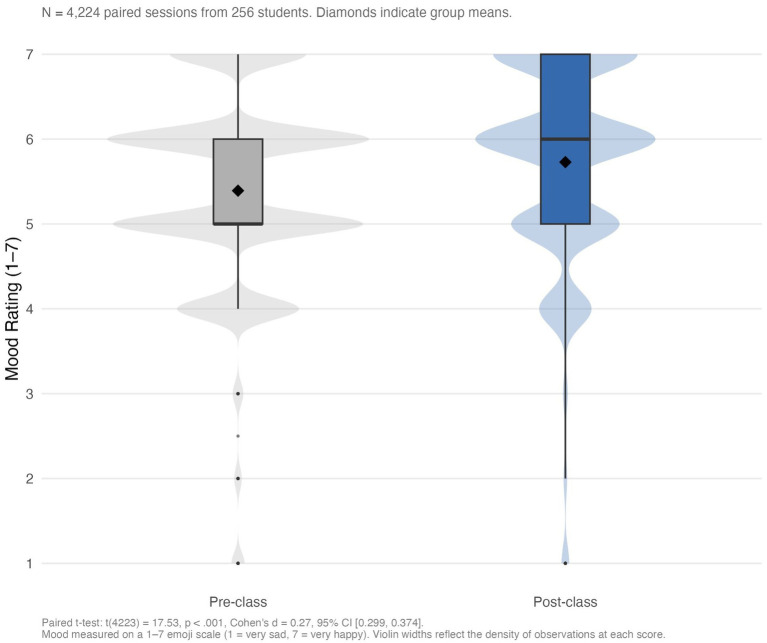
Session-level mood before and after dance class. Violin plots with embedded box plots display the distribution of pre-class (grey) and post-class (blue) mood ratings across 4,224 paired sessions from 256 students. Diamonds indicate group means. Pre-class mood averaged 5.39 (SD = 1.19) and post-class mood averaged 5.73 (SD = 1.20), a mean gain of 0.34 points (6.2% improvement). The difference was statistically significant, *t*(4223) = 17.53, *p* < 0.001, Cohen’s d = 0.27, 95% CI of change [0.299, 0.374]. The prominent spike at the scale ceiling (7) in post-class ratings illustrates the right-censoring addressed by the Tobit sensitivity analysis (Section 3.3). Mood was measured on a 1–7 emoji scale (1 = very sad, 7 = very happy). Violin widths reflect the density of observations at each score.

*Overall Mood Change (Primary Outcome).* Across 4,224 paired class sessions from 256 students, pre-class mood averaged 5.39 (*SD* = 1.19) and post-class mood averaged 5.73 (*SD* = 1.20), yielding a mean increase of 0.34 points (*SD* = 1.25). The session-level paired *t*-test was significant, *t*(4223) = 17.53, *p* < 0.001, Cohen’s *d* = 0.27, 95% CI [0.299, 0.374]. This corresponds to roughly a 6.2% improvement from baseline mood as shown in Figure 2.

**Figure 3:**
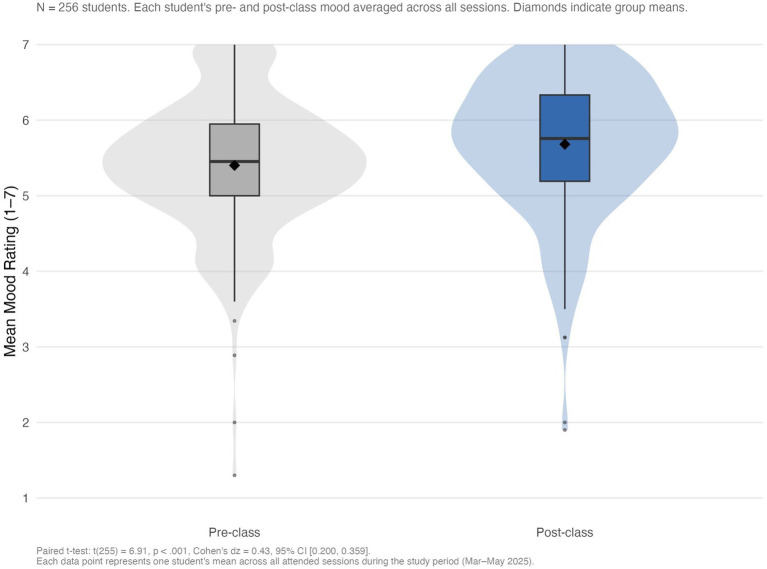
Per-student averaged mood before and after dance class. Violin plots with embedded box plots display the distribution of each student’s mean pre-class (grey) and mean post-class (blue) mood, averaged across all sessions attended during the study period (February 19–May 31, 2025). *N* = 256 students. Diamonds indicate group means. Per-student pre-class mood averaged 5.40 and post-class averaged 5.68, a mean gain of 0.28 points, *t*(255) = 6.91, *p* < 0.001, Cohen’s d_z_ = 0.43, 95% CI [0.20, 0.36]. This student-level analysis complements the session-level results in Figure 2 by treating each student as a single data point regardless of how many sessions they contributed, ensuring that prolific attendees do not disproportionately influence the estimate.

A per-student analysis that first averaged each student’s sessions confirmed the pattern. Across 256 students, per-student pre-class mood averaged 5.40 and post-class averaged 5.68, a mean gain of 0.28 points. The paired *t*-test was significant, *t*(255) = 6.91, *p* < 0.001, Cohen’s *d*_z = 0.43. The larger per-student effect size reflects reduced within-student noise after aggregation as shown in Figure 3.

### 3.2 Multilevel models

Because each student contributed a variable number of sessions (*M* = 16.5, *Mdn* = 9, range 1–109), we used multilevel models as the primary inferential framework. The analytic sample comprised 4,059 sessions from 251 students after excluding rare class types (Tap, Other) and listwise deletion of records with missing covariates.

*Variance partitioning.* The null model (M0) yielded an ICC of 0.432, indicating that 43.2% of the variance in post-class mood reflected stable between-student differences rather than within-session fluctuations. This substantial clustering confirmed the need for multilevel modeling as shown in Table 1.

**Table 1:** Multilevel model comparison for post-class mood.

Model	Description	AIC	BIC	*R*^2^m	*R*^2^c	LR test
M0	Null (intercept only)	1,669	1,688	—	0.432	—
M1	+ Pre-mood (centered)	1,331	1,357	0.082	0.386	*p* < 0.001 vs. M0
M2	+ Frequency cohort	1,338	1,375	0.088	0.390	*p* = 1.0 vs. M1
M3	+ All covariates	1,424	1,588	0.095	0.397	*p* = 1.0 vs. M2
M4	+ Random slopes (pre-mood)	1,223	1,399	0.104	0.462	*p* < 0.001 vs. M3
M5	+ Instructor random effect	1,424	1,595	0.096	0.399	*p* = 0.276 vs. M3

Sequential model-building results showing five nested models predicting post-class mood (1–7 emoji scale) from 4,059 sessions from 251 students (analytic sample after excluding rare class types and listwise deletion of records with missing covariates). M0 = null model (random intercept only, ICC = 0.432); M1 = pre-class mood added as Level 1 covariate (group-mean centered); M2 = frequency cohort added as Level 2 predictor (1–3/week reference); M3 = class type, proficiency level, time of day, day of week, and instructor experience added; M4 = random slope for pre-class mood added (final model); M5 = instructor added as crossed random effect (not retained). Each row reports AIC, BIC, likelihood ratio test against the indicated comparison model, marginal *R*^2^ (variance explained by fixed effects alone), and conditional *R*^2^ (variance explained by fixed and random effects combined). *R*^2^ values computed via the Nakagawa and Schielzeth (2013) method. M4 was selected as the best-fitting model based on AIC reduction and theoretical parsimony. See Section 3.2 for full results.

**Figure 4:**
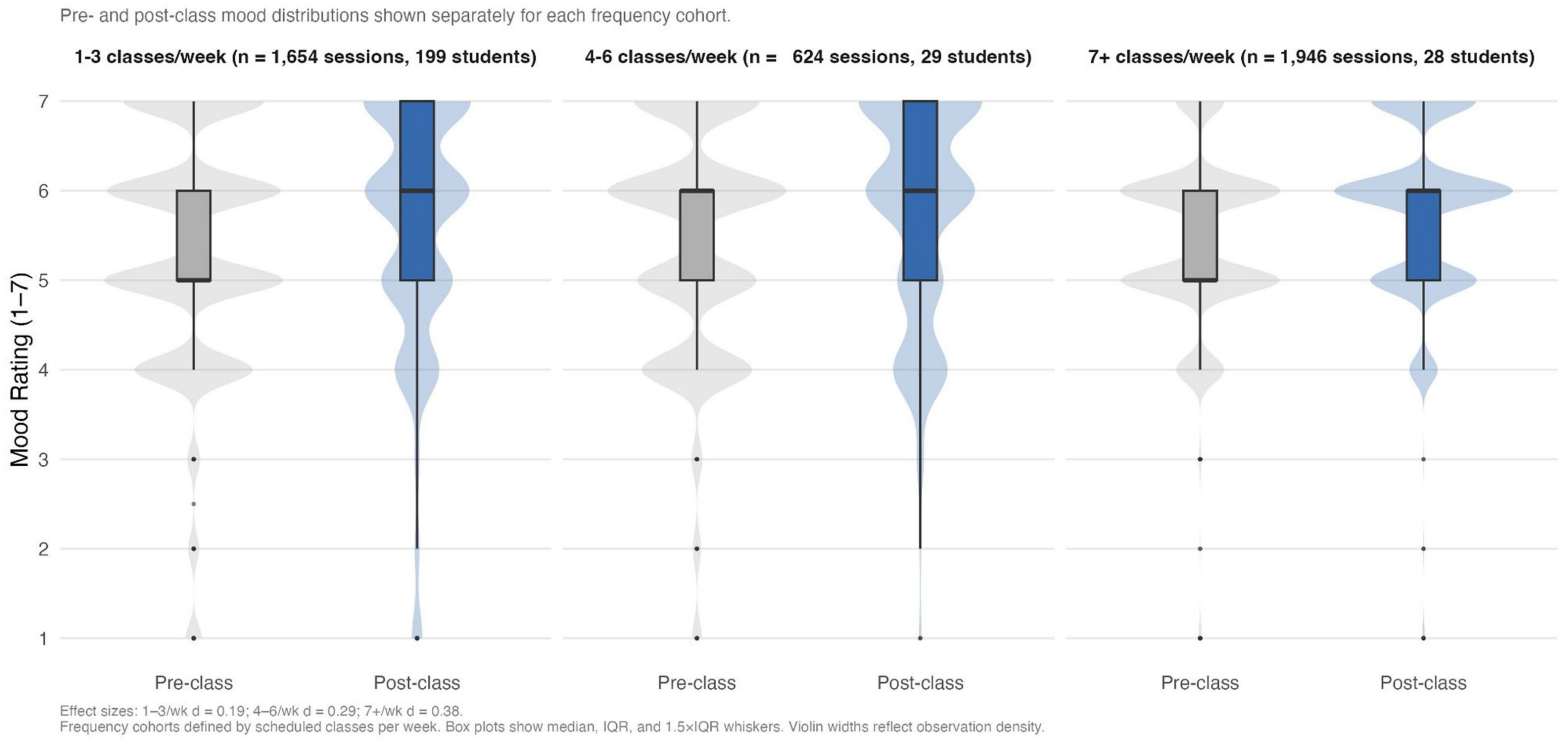
Pre- and post-class mood distributions by weekly dance class frequency. Violin plots with embedded box plots are shown separately for three frequency cohorts: 1–3 classes/week (*n* = 1,654 sessions, 199 students), 4–6 classes/week (*n* = 624 sessions, 29 students), and 7 + classes/week (*n* = 1,946 sessions, 28 students). Gray violins represent pre-class mood; blue violins represent post-class mood. Box plots show median, interquartile range, and 1.5 × IQR whiskers. Session-level effect sizes: 1–3/week *d* = 0.19, 4–6/week *d* = 0.29, 7+/week *d* = 0.38. Despite this descriptive gradient, frequency cohort was not a statistically significant predictor of post-class mood in the multilevel model after controlling for pre-class mood (Section 3.2), nor in the Tobit sensitivity analysis (Section 3.3). The non-significant finding may reflect selection effects: students who attend more classes may differ systematically from those who attend fewer.

*Model-building results.* Table 1 summarizes the sequential model comparisons. Adding pre-class mood as a Level 1 covariate (M1) substantially improved fit over the null model. Pre-class mood was the strongest predictor across all models (M1: *B* = 0.28, *SE* = 0.014, *p* < 0.001). Weekly frequency cohort (M2) did not significantly improve model fit (*p* = 1.0), with neither the 4–6/week (*B* = 0.13, *p* = 0.355) nor the 7+/week cohort (*B* = 0.18, *p* = 0.198) differing significantly from the 1–3/week reference group as shown in Figure 4.

The full covariate set in M3, including class type, proficiency level, time of day, day of week, and instructor experience, likewise failed to improve fit beyond M2 (likelihood ratio *p* = 1.0). No individual covariate reached significance.

*Random slopes.* Allowing the pre-class mood slope to vary across students (M4) produced the largest single improvement in model fit (ΔAIC = 201, likelihood ratio *p* < 2.2e-16). This indicates that students differed substantially in how their pre-class mood related to post-class mood: for some students, a low pre-class mood strongly predicted a large mood gain; for others, the association was weaker. Adding instructor as a crossed random effect (M5) did not improve fit (*p* = 0.276), confirming that instructor-level variation in mood outcomes was negligible.

*Summary.* M4 (random slopes, no instructor effect) was the best-fitting model. Marginal *R*^2^ was 0.104 (variance explained by fixed effects alone) and conditional *R*^2^ was 0.462 (variance explained by both fixed and random effects). The large gap between marginal and conditional *R*^2^ underscores that individual differences, not observable session-level covariates, drove most of the systematic variation in post-class mood.

### 3.3 Sensitivity analysis: ceiling effects

Of 4,059 post-class mood ratings in the analytic sample, 28.8% reached the scale maximum (7 on a 1–7 scale). To assess whether this right censoring biased estimates, we fit Tobit regression models with cluster-robust standard errors.

The Tobit model revealed that ceiling compression substantially attenuated the pre-class mood effect. The pre-mood coefficient approximately doubled, from 0.28 in the MLM to 0.63 in the Tobit model, suggesting that the true relationship between entering mood and leaving mood was considerably stronger than standard linear models indicated.

Frequency cohort remained non-significant in the Tobit framework (4–6/week: *B* = 0.26, *p* = 0.202; 7+/week: *B* = 0.18, *p* = 0.518), and the frequency × pre-class mood interaction was not significant (*p* = 0.823 and 0.803), indicating that ceiling compression did not differentially mask frequency effects.

One class-type finding emerged in the Tobit model that was not apparent in the MLM: Pointe classes were associated with significantly lower post-class mood (*B* = −0.28, *p* < 0.001). Pointe students (*n* = 24 students, 340 sessions) did not enter class with elevated mood (pre-class *M* = 5.29, comparable to other genres), ruling out a simple ceiling explanation. A more likely interpretation is that Pointe is the most physically demanding and painful form of dance, students perform on the tips of their toes for extended periods, and this discomfort may attenuate the mood gains observed in other genres. The mood change for Pointe sessions was still positive (*Δ* = 0.28) but smaller than Ballet (0.42), Jazz (0.39), or Hip Hop (0.42). Given the relatively small number of Pointe students, this finding should be treated as exploratory.

### 3.4 Ceiling-aware mood trajectories

**Figure 5:**
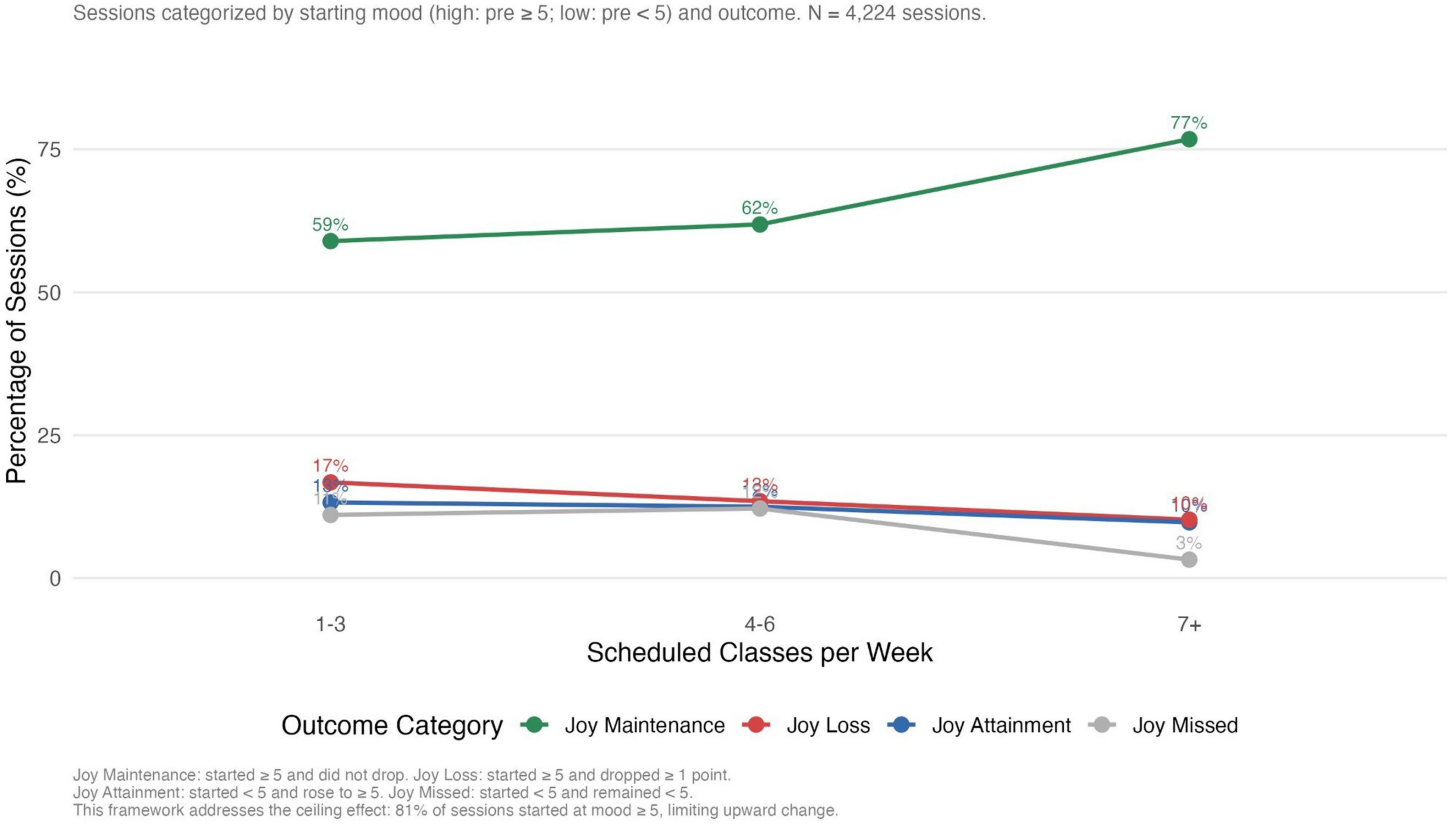
Ceiling-aware mood outcomes by weekly dance class frequency. Each session was classified into one of four mutually exclusive categories based on starting mood and outcome: Joy maintenance (started ≥ 5 and did not drop), joy loss (started ≥ 5 and dropped ≥ 1 point), joy attainment (started < 5 and rose to ≥ 5), and joy missed (started < 5 and remained < 5). This framework addresses the 28.8% ceiling rate by distinguishing maintained high mood from upward change. *N* = 4,224 sessions. Joy maintenance was the dominant outcome across all cohorts and increased descriptively with frequency (59% at 1–3/week, 62% at 4–6/week, 77% at 7+/week). Joy loss decreased from 17 to 10%. These patterns are descriptive; the multilevel model did not find a statistically significant effect of frequency cohort on post-class mood (Section 3.2).

To characterize mood patterns beyond mean change, we classified each session into four outcome categories based on whether mood started above or below the scale midpoint (see Section 2.6.3). Among the three frequency cohorts, descriptive patterns were consistent with greater mood stability at higher participation levels, though these differences did not reach significance in the multilevel models reported above. In the 1–3/week cohort (*n* = 1,654 sessions from 199 students), 58.9% of sessions were classified as Joy Maintenance and 16.7% as Joy Loss. In the 4–6/week cohort (*n* = 624 sessions from 29 students), 61.9% were Joy Maintenance and 13.5% Joy Loss. In the 7+/week cohort (*n* = 1,946 sessions from 28 students), 76.8% were Joy Maintenance and 10.2% Joy Loss as shown in Figure 5.

These descriptive patterns suggest a trend toward stronger mood stability among more frequent dancers, but given the non-significant frequency effect in the MLM, selection effects cannot be ruled out: students who attend more frequently may differ systematically from less frequent attenders in ways that predispose them to higher and more stable mood.

### 3.5 Exploratory patterns

**Figure 6:**
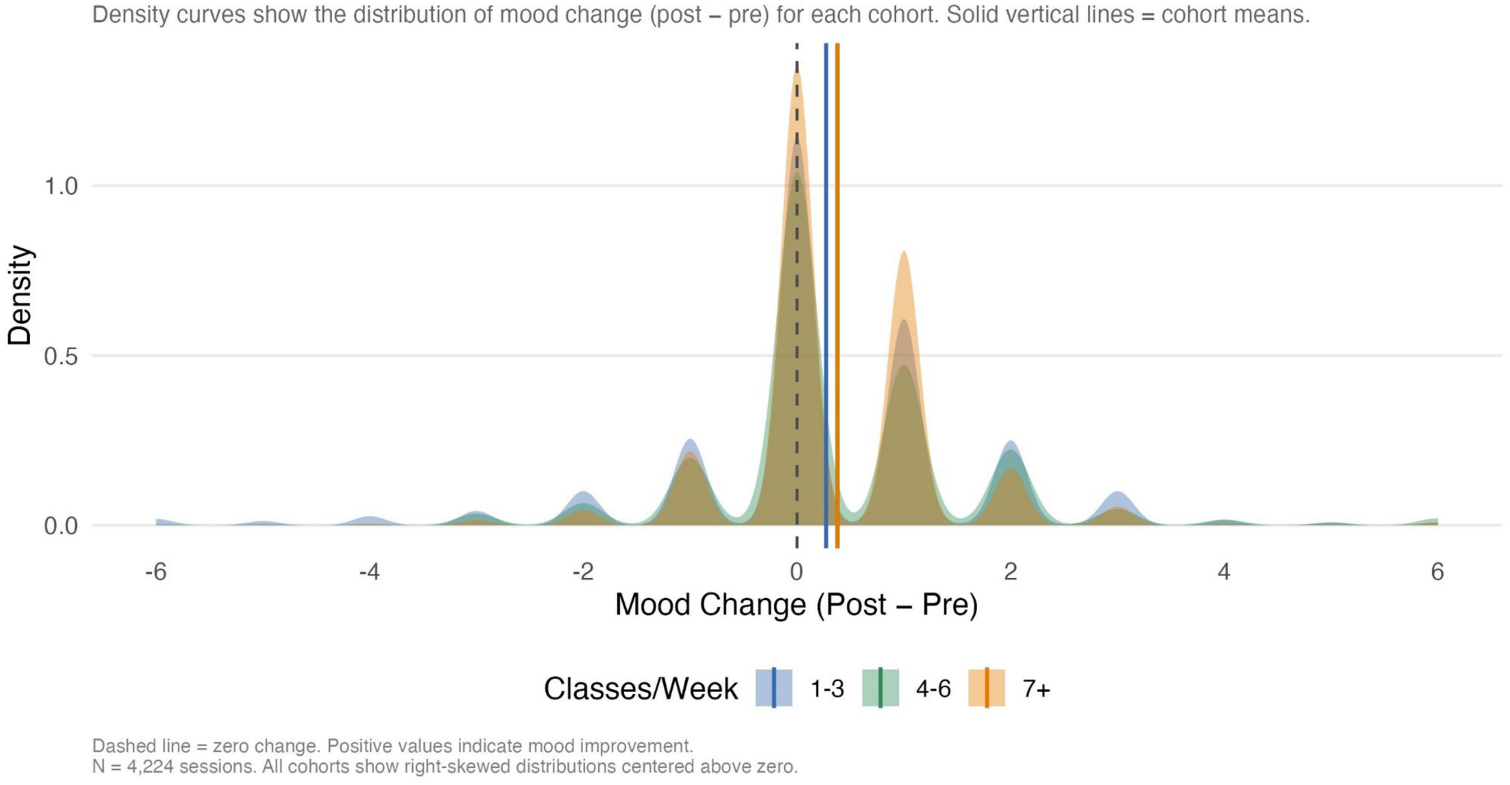
Session-level mood change by dance genre. Horizontal violin plots with embedded box plots display the distribution of within-session mood change (post minus pre) across seven dance genres. Diamonds indicate means; dashed vertical line indicates zero change. Genres are ordered by median mood change. *N* per genre: Ballet = 1,311; Combo = 952; Hip Hop = 393; Jazz = 571; Contemporary/Lyrical = 362; Company Rehearsal = 130; Pointe = 340. Tap was excluded (*n* = 15 sessions). Class type was not a statistically significant predictor in the multilevel model (Section 3.2). The Tobit sensitivity analysis identified one significant class-type effect: Pointe was associated with lower post-class mood (*B* = −0.28, *p* < 0.001), potentially reflecting the demanding technical nature of Pointe work among advanced students who also exhibited higher pre-class mood (Section 3.3).

*Baseline mood.* Pre-class mood was the strongest predictor of post-class change across all analyses. Sessions starting at low mood (1–5) improved 56.6% of the time and declined in only 7.3%, whereas sessions starting at high mood (6–7) improved 20.1% and declined 20.6%. This regression-to-the-mean pattern is expected given the bounded scale and was confirmed by the substantial pre-mood coefficient in both the MLM (*B* = 0.28) and Tobit (*B* = 0.63) models as shown in Figure 6.

**Figure 7:**
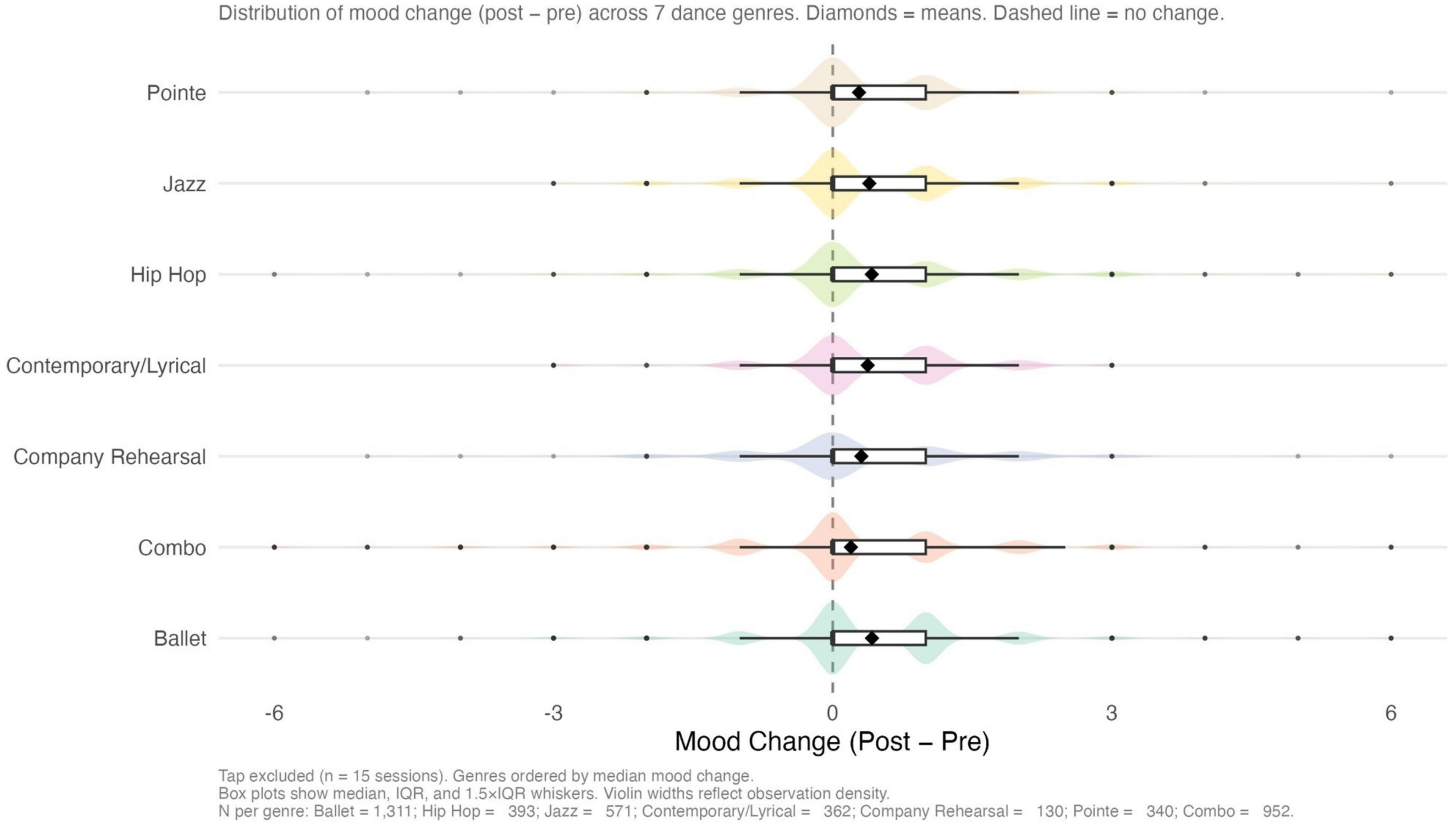
Session-level mood change by student proficiency level. Violin plots with embedded box plots display the distribution of within-session mood change (post minus pre) across four instructor-assigned proficiency levels. Diamonds indicate means; dashed horizontal line indicates zero change. N per level: Beginner = 686 sessions (98 students), Intermediate = 1,284 sessions (98 students), Advanced = 1,874 sessions (54 students), Expert = 380 sessions (6 students). All proficiency groups showed mean mood improvements above zero with overlapping distributions. Proficiency was not a statistically significant predictor of post-class mood in the multilevel model (Section 3.2), consistent with the interpretation that acute mood benefits of dance are broadly distributed across skill levels.

*Class type*. Descriptive analyses showed mood improvement rates ranging from 31.4% (Pointe) to 45.9% (Company Rehearsal). However, class type was not a significant predictor in the multilevel model, and the Tobit model identified only Pointe as significant (and negative). We interpret this pattern as evidence that the acute mood benefit of dance generalizes across genres rather than being contingent on any particular style as shown in Figure 7.

**Figure 8:**
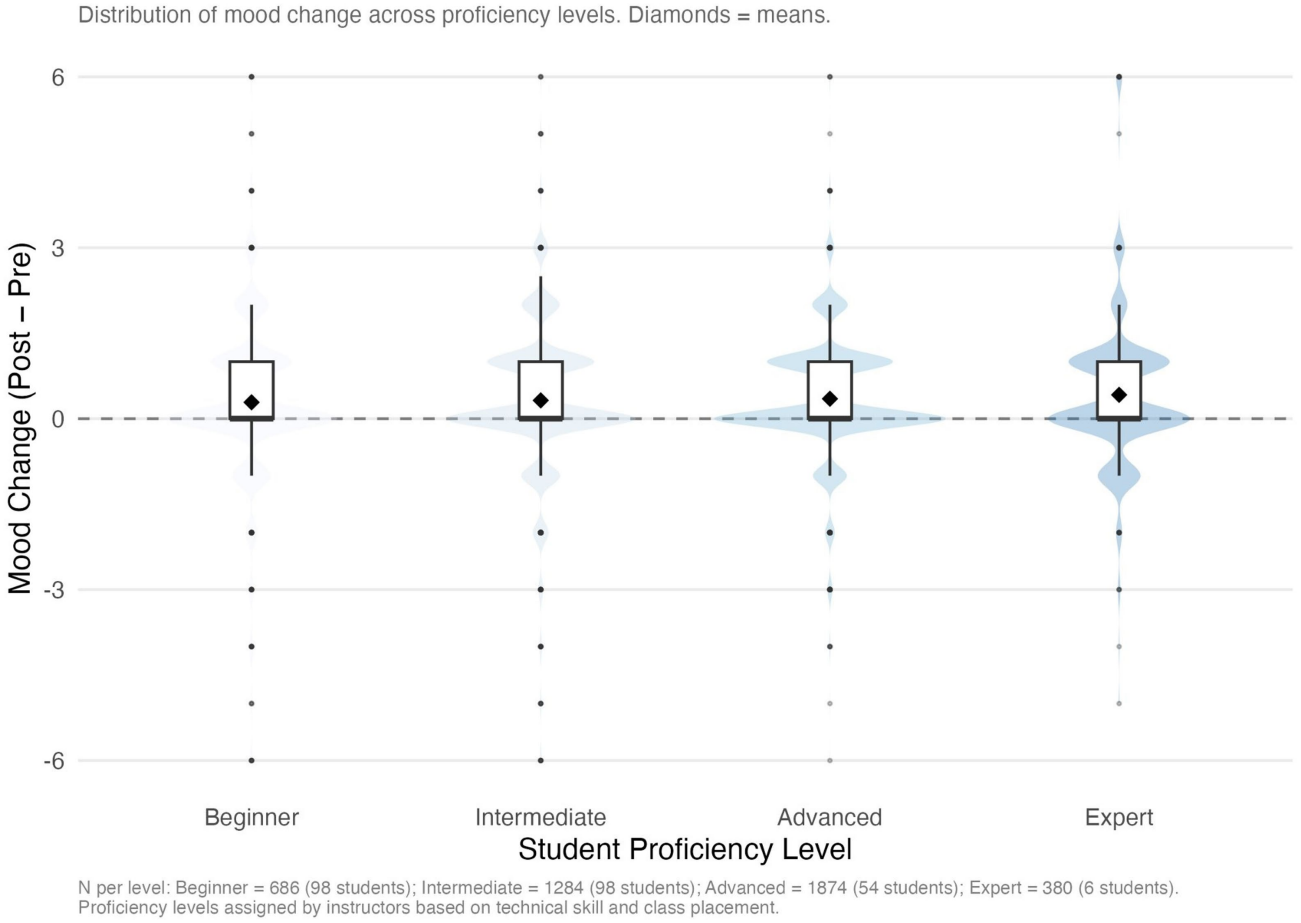
Distribution of session-level mood change by frequency cohort. Density curves show the distribution of within-session mood change (post minus pre) for each frequency cohort: 1–3 classes/week (blue), 4–6 classes/week (green), and 7 + classes/week (orange). Solid vertical lines indicate cohort means; dashed line indicates zero change. *N* = 4,224 sessions. All three cohorts display right-skewed distributions centered above zero, indicating that mood improvements were more common than declines regardless of attendance frequency. Positive values indicate mood improvement; negative values indicate decline.

*Time and scheduling.* Late-afternoon sessions yielded the highest improvement rate (40.7%), and mid-week sessions (Tuesday through Thursday) showed slightly higher rates than Monday or Friday. Neither time of day nor day of week reached significance in the MLM, suggesting these descriptive patterns are small in magnitude relative to individual differences as shown in Figure 8.

**Figure 9:**
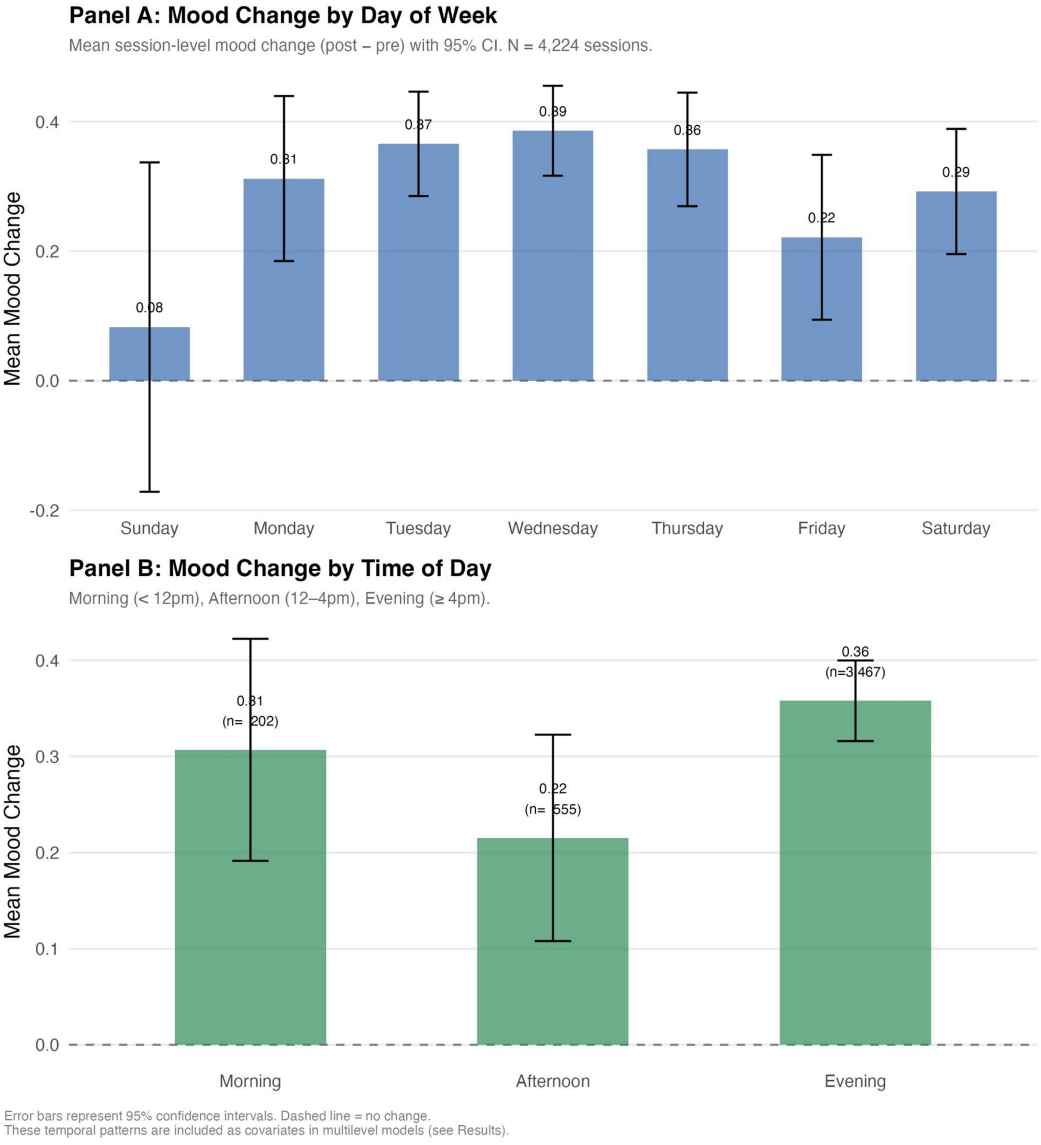
Temporal patterns in mood change. **Panel A**: Mean session-level mood change (post minus pre) by day of week with 95% confidence intervals. *N* = 4,224 sessions. Mid-week sessions (Tuesday through Thursday) showed the highest mean gains (0.36–0.39), while Sunday showed the smallest (0.08). **Panel B**: Mean mood change by time of day—morning (before 12:00 p.m., *n* = 201), afternoon (12:00–4:00 p.m., *n* = 555), and evening (4:00 p.m. or later, *n* = 3,467). Evening sessions showed the largest mean change (0.36). Error bars represent 95% confidence intervals. Dashed lines indicate zero change. Neither day of week nor time of day reached statistical significance as predictors in the multilevel model after controlling for pre-class mood (Section 3.2). These temporal variables were included as covariates in the multilevel models, as recommended by Reviewer 4.

**Proficiency and experience.** All proficiency groups (Beginner through Expert) showed significant pre-to-post mood gains in session-level analyses. The MLM confirmed that proficiency was not a significant predictor of post-class mood, nor was instructor experience level. Mood benefits were evident regardless of student skill level or instructor seniority as shows in Supplementary Figure S1, Figure 9.

### 3.6 Convergent and discriminant validity

**Figure 10:**
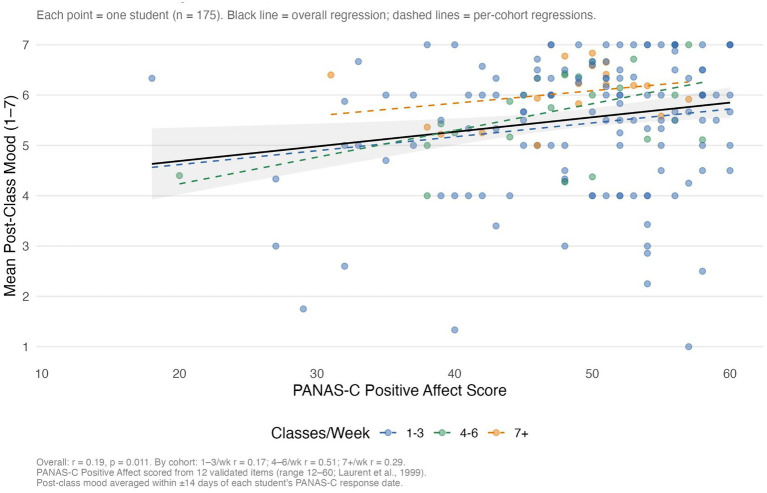
Convergent validity: PANAS-C positive affect and mean post-class emoji mood. Each point represents one student (*n* = 175 students who completed PANAS-C within ±14 days of at least one mood-assessed session). The *x*-axis shows PANAS-C positive affect scores (12 validated items; range 12–60; Laurent et al., 1999). The *y*-axis shows each student’s mean post-class mood rating during the ±14-day window. The solid black line is the overall regression (*r* = 0.19, *p* = 0.011). Dashed colored lines show per-cohort regressions: 1–3/week (blue, *r* = 0.17), 4–6/week (green, *r* = 0.51), 7+/week (orange, *r* = 0.29). The association was statistically significant overall and for the 1–3/week and 4–6/week cohorts. The modest overall magnitude likely reflects the well-established distinction between momentary state affect (emoji scale) and dispositional trait affect (PANAS-C), with stronger convergence among students who provided more sessions during the matching window.

To assess whether the single-item emoji mood scale captured meaningful affective variance, we correlated each student’s mean post-class mood rating with their PANAS-C Positive Affect score. Among the 175 students with both complete PANAS-C data and mood sessions within the ±14-day window, the overall correlation was modest but statistically significant (*r* = 0.19, *p* = 0.011, 95% CI [0.04, 0.33]). To evaluate discriminant validity, we also correlated mean post-class mood with PANAS-C Negative Affect for the same sample. As expected, this correlation was small and non-significant (*r* = −0.06, *p* = 0.422, *n* = 175), indicating that the emoji mood scale tracked positive affective content specifically rather than general response tendency or undifferentiated affect as shown in Figure 10.

**Figure 11:**
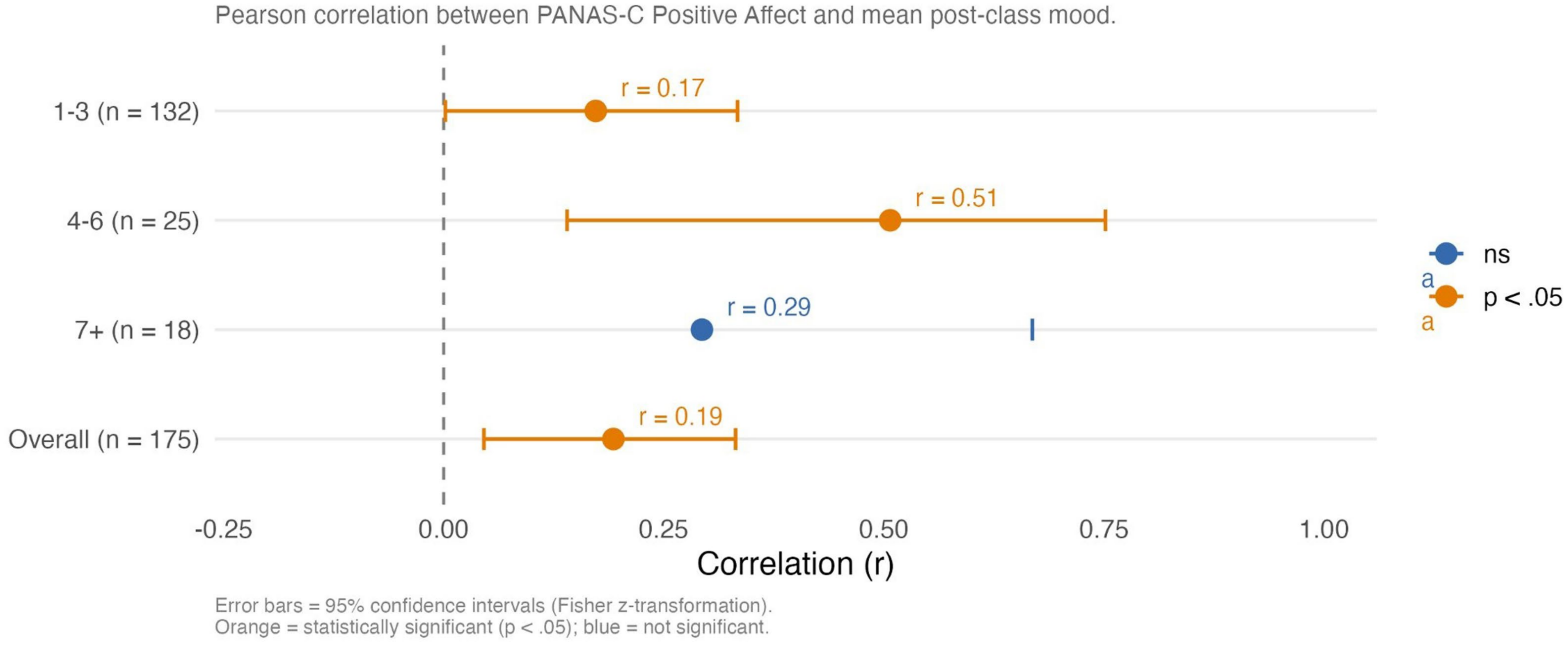
Convergent validity by frequency cohort (forest plot). Pearson correlations between PANAS-C Positive Affect score and mean post-class emoji mood, with 95% confidence intervals calculated via Fisher z-transformation. Orange points indicate statistically significant correlations (*p* < 0.05); blue points indicate non-significant correlations. Cohort sample sizes: 1–3/week (*n* = 132), 4–6/week (*n* = 25), 7+/week (*n* = 18), Overall (*n* = 175). Cohort correlations: 1–3/week r = 0.17, 4–6/week r = 0.51, 7+/week r = 0.29 (ns). The pattern of stronger convergence among more frequent attendees is consistent with greater measurement reliability when more sessions contribute to each student’s mean. However, the 4–6/week and 7+/week confidence intervals are wide due to small subgroup sample sizes and should be interpreted cautiously.

Stratified by frequency cohort, the association was significant for 1–3/week and 4–6/week students, with the strongest correlation at moderate frequency 1–3/week (*n* = 132, *r* = 0.17, *p* = 0.048), 4–6/week (*n* = 25, *r* = 0.51, *p* = 0.010), and 7+/week (*n* = 18, *r* = 0.29, *p* = 0.237). The peak association at 4–6 classes per week is consistent with a nonlinear dose–response pattern in which moderate participation frequency optimizes alignment between momentary and dispositional affect. The small subgroup sizes for 4–6/week and 7+/week cohorts limit the precision of these stratified estimates as shown in Figure 11.

These results provide evidence for convergent validity between the emoji mood scale and PANAS-C Positive Affect. The modest overall magnitude likely reflects the difference between a momentary state measure (post-class mood captured immediately after a specific class) and a dispositional trait measure (PANAS-C Positive Affect reflecting general affective tendencies over the past few weeks). The strongest association at 4–6 classes per week suggests that moderate-frequency participants may develop a sufficiently regular affective response to dance that their post-class mood better approximates their dispositional affect, whereas less frequent dancers show greater session-to-session variability and the most frequent dancers (7+/week) did not show a significant association (*r* = 0.29, *p* = 0.237), possibly reflecting ceiling or fatigue effects that attenuate the state–trait correspondence. This interpretation is speculative given the small subgroup sizes and cross-sectional nature of the PANAS-C administration.

## 4 Discussion

Given the growing stressors facing youth, from excessive screen time and social media pressure to climate anxiety and AI-driven career uncertainty, there is a critical need for preventive, community-based strategies that support mood regulation. Our data suggest that structured dance participation may serve such a function. Across more than 4,000 class sessions, students reliably left class in better mood than they entered, a pattern that held regardless of dance genre, proficiency level, instructor experience, or time of day. The consistency of this benefit across observable covariates, rather than its magnitude, may be the most practically significant finding.

### 4.1 Summary of key findings

Three findings emerged from the multilevel and sensitivity analyses. First, dance classes were associated with a reliable acute mood benefit: post-class mood was significantly higher than pre-class mood across the full sample (session-level *d* = 0.27; per-student *d*_z = 0.43), and the multilevel model confirmed this after accounting for the nested data structure (ICC = 0.432).

Second, the strongest predictor of post-class mood was pre-class mood, not any observable characteristic of the student, class, or instructor. No covariate, including weekly frequency, class type, proficiency level, time of day, or instructor experience, reached significance in the multilevel model. The Tobit sensitivity analysis confirmed that this null pattern was not an artifact of ceiling compression: frequency remained non-significant even after adjusting for the 28.8% of sessions at the scale maximum. The single exception was Pointe, which showed a significant negative coefficient in the Tobit model (*B* = −0.28, *p* < 0.001), likely reflecting the physical pain inherent to dancing en pointe.

Third, individual differences dominated. The random-slopes model (M4) dramatically improved fit (ΔAIC = 201), revealing that students varied substantially in how their entering mood translated to post-class mood. The gap between marginal *R*^2^ (0.104) and conditional *R*^2^ (0.462) confirmed that who the student was mattered far more than what class they attended or how often they attended it.

### 4.2 Interpretation in context

The present findings situate dance alongside other established exercise and arts-based interventions for mood regulation, while clarifying what dance does and does not do. The acute mood benefit (Cohen’s *d* = 0.27) falls squarely within the range of published effect sizes for comparable youth programs: physical education interventions (*d* ≈ 0.20–0.40; Biddle and Asare, 2011), mindfulness programs (*d* ≈ 0.23; Klingbeil et al., 2017), and social–emotional learning curricula (*d* ≈ 0.25; Durlak et al., 2011). What distinguishes the present study is its ecological validity, these effects were observed in naturalistic dance classes, not controlled laboratory sessions, and sustained across thousands of repeated measurements.

The null finding for frequency deserves careful interpretation. The multilevel models found no significant difference in post-class mood across the 1–3, 4–6, and 7 + classes/week cohorts. This does not mean frequency is unrelated to mood; it means that within the multilevel framework, frequency did not explain additional variance beyond pre-class mood and individual random effects. The descriptive ceiling-aware metrics showed a suggestive gradient, Joy Maintenance increased from 58.9% at 1–3/week to 76.8% at 7+/week, but this pattern is confounded with selection: students who attend more frequently may differ from less frequent attenders in temperament, family support, or intrinsic motivation, all of which could independently predict higher mood stability.

H3, predicting stronger mood benefits at higher participation frequencies, was not supported by the multilevel models. Although broaden-and-build theory (Fredrickson, 2001) posits that repeated positive affective experiences accumulate into enduring personal resources, the current design cannot test this mechanism: the null frequency finding could reflect either a true absence of dose–response effects or the confounding of attendance frequency with unmeasured dispositional and family variables. An experimental design with random assignment to frequency conditions would be needed to isolate the effect.

The finding that individual differences drove most systematic variance is itself informative. If the acute mood benefit of dance were driven primarily by genre, instructor skill, or scheduling, we would expect the full-covariate model (M3) to substantially outperform the pre-mood-only model (M1). It did not. This suggests that the mood-lifting property of dance is robust and general, a feature of the activity itself rather than its specific delivery parameters. For practitioners, this is encouraging: any well-run dance class appears to carry the benefit, without requiring a specific genre or time slot.

### 4.3 Convergent validity and measurement

The overall correlation between the single-item emoji mood scale and PANAS-C Positive Affect (*r* = 0.19, *p* = 0.011) provides evidence for convergent validity, though the magnitude is modest. This is consistent with the theoretically expected pattern: the emoji scale captured momentary post-class affect (a state), while the PANAS-C assessed general affective tendencies over the past few weeks (a trait). State–trait correlations are typically modest because transient fluctuations in state affect are only partially explained by dispositional tendencies (Spielberger, 1972; Watson and Clark, 1994).

The frequency-stratified correlations revealed a nonlinear pattern (1–3/week: *r* = 0.17; 4–6/week: *r* = 0.51; 7+/week: *r* = 0.39), with the strongest state–trait correspondence at moderate participation frequency. This pattern is consistent with the broader physical activity literature, where psychological benefits often follow an inverted-U dose–response curve rather than a linear increase (Ekkekakis, 2003; Hamer and Stamatakis, 2010). Students attending 4–6 classes per week may accumulate sufficient regularity for their post-class mood to stabilize and align with dispositional affect, while the most frequent dancers (7+/week, predominantly competitive or pre-professional) may experience training fatigue, performance pressure, or habituation effects that attenuate the state–trait correspondence. However, this interpretation is speculative, and the small subgroup sizes (particularly *n* = 23 and *n* = 26 for the higher-frequency cohorts) limit the precision of these stratified estimates.

### 4.4 Practical implications

*For educators and program designers.* The finding that no covariate moderated the acute mood benefit has a practical upshot: dance studios need not optimize for a specific genre, time slot, or instructor profile to achieve mood gains. The benefit appears general. Studios should ensure consistent, well-structured classes and recognize that mood enhancement may be an inherent property of dance participation rather than a feature that requires engineering.

The Pointe finding suggests a nuance: physically painful genres may attenuate, but not eliminate, the mood benefit. This does not argue against Pointe instruction, but it does suggest that instructors should be mindful of the affective toll of pain and consider whether warm-up or cool-down rituals could buffer the discomfort.

*For parents, communities, and policy-makers.* These findings position dance as a viable component of community-based mental health strategy, particularly for youth. The observed effect size (*d* = 0.27), while modest in absolute terms, operates at scale: a child attending three dance classes per week accumulates over 150 mood-positive experiences per year. The practical significance of such repeated low-intensity benefits should not be underestimated in the context of preventive mental health (Kazdin and Blase, 2011).

### 4.5 Limitations

Several limitations constrain interpretation. First, the observational design precludes causal attribution. Without a randomized control group, the observed pre-to-post mood improvements could reflect regression to the mean, demand characteristics (students knowing their mood was being tracked), social desirability, or the natural passage of time during class rather than dance-specific effects. The bounded 1–7 scale, combined with a starting average of 5.39, makes regression toward the mean particularly plausible for sessions beginning below the midpoint.

Second, the sample was drawn from a single studio system in one metropolitan area, with a predominantly female, non-clinical population. Generalizability to boys, clinical populations, community recreation programs, or different cultural contexts is unknown.

Third, the single-item emoji mood scale, while practical for repeated administration in a youth dance setting, lacks the psychometric depth of multi-item validated instruments. The convergent validity with the PANAS-C (*r* = 0.19), while statistically significant, indicates that the emoji scale captured some but not all affective variance that multi-item measures would detect. Habituation effects are also possible: students completing mood ratings before and after every class may have developed routine response patterns that compressed true variance.

Fourth, the PANAS-C was administered cross-sectionally rather than longitudinally, precluding assessment of whether dance participation was associated with trait-level affective changes over time. The convergent validity analysis was therefore limited to a concurrent association between state and trait measures.

Fifth, selection bias is a core limitation. Families who invest in structured dance training may already provide enriched environments that support emotional well-being. The non-significant frequency effect in the MLM could reflect either a true absence of dose–response or a confounding of attendance frequency with unmeasured family and temperament variables.

Sixth, the researcher–practitioner overlap in this study (co-investigators were affiliated with the studio system) introduces potential conflicts of interest. Although mood data were collected via automated digital tools with minimal investigator contact, the absence of independent data collection should be noted.

### 4.6 Future directions

Several planned extensions address the limitations identified above. A follow-up study currently in preparation will expand data collection across multiple studio sites and community partner organizations, including partnerships with shelters, Head Start programs, and community centers, to test whether the mood benefits observed here generalize beyond the predominantly female, middle-to-upper socioeconomic sample of the present study. This multi-site design will also incorporate quarterly PANAS-C administration with 6- and 12-month follow-ups, permitting a direct test of whether repeated dance participation is associated with trait-level affective changes rather than only transient state improvements.

Critically, the follow-up study includes a randomized controlled trial comparing emoji-based mood scales with standard 7-point Likert ratings, using equivalence testing (Two One-Sided Tests) to evaluate whether simplified visual formats produce statistically equivalent pre–post mood change estimates. This addresses a central limitation of the present work: the single-item emoji scale showed modest convergent validity with the PANAS-C (r = 0.19), and the field lacks experimental evidence on whether pictorial and numeric mood formats yield comparable data in youth. The RCT employs both parallel (fixed-format) and crossover (rotating-format) arms, enabling assessment of habituation effects and spatial response bias alongside format equivalence. All primary analyses will be pre-registered.

Beyond measurement, the most promising substantive question raised by the present findings concerns individual differences. The random slopes model (ΔAIC = 201) indicates that students vary substantially in how their pre-class mood translates to post-class mood. Understanding what makes some students more responsive to dance, temperamental factors, social context, developmental stage, or program features not captured here, could inform targeted programming and is a priority for future investigation. Designs that include waitlist or no-activity control conditions would additionally permit causal inference about dance-specific effects, separating the activity’s contribution from regression to the mean and social demand.

## Data Availability

The deidentified analytic dataset and full reproducible R analysis pipeline supporting the conclusions of this study are publicly available on GitHub at https://github.com/phenlab/dance-mood-replication. The repository includes all preparation, scoring, validation, and modeling scripts (scripts/ directory, executed sequentially via run_all.R), the deidentified session-level and student-level analytic data (data_deid/), and exact R package versions (renv.lock) to support full computational reproducibility. Raw, unredacted participant data are not publicly available due to IRB restrictions protecting minor participants. Reasonable requests for additional information should be directed to the corresponding author.

## Supplementary material

SUPPLEMENTARY FIGURE S1 Session-level mood change by instructor experience level. Violin plots with embedded box plots display the distribution of within-session mood change (post minus pre) across four management-assigned instructor experience levels. Diamonds indicate means. N per level: Beginner = 141 sessions, Intermediate = 1,360 sessions, Advanced = 982 sessions, Expert = 1,741 sessions. All instructor experience groups showed comparable mean mood improvements with overlapping distributions. Instructor experience was not a statistically significant predictor of post-class mood in the multilevel model (M5, Section 3.2). This figure is included as supplementary material because the variable did not improve model fit and was not retained in the final model.
