# Supplementary figures and images for: Mood changes of children and adolescents in dance classes: a prospective repeated-measures study
Source: Front Psychol. 2026 Jun 11;17:1719704. doi: 10.3389/fpsyg.2026.1719704 (PMC13293843; doi:10.3389/fpsyg.2026.1719704)

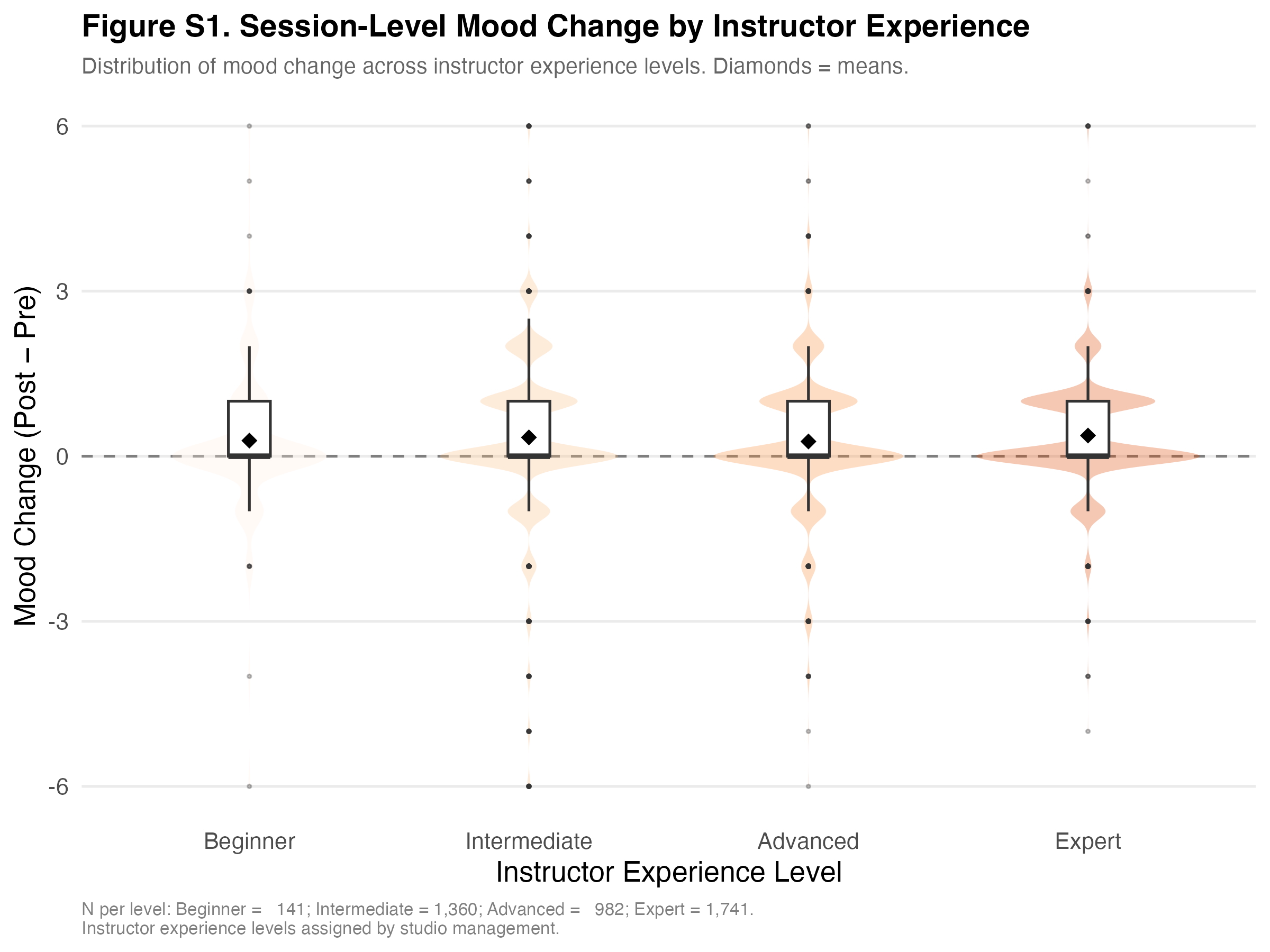

Supplement: SUPPLEMENTARY FIGURE S1 — Session-level mood change by instructor experience level. Violin plots with embedded box plots display the distribution of within-session mood change (post minus pre) across four management-assigned instructor experience levels. Diamonds indicate means. N per level: Beginner = 141 sessions, Intermediate = 1,360 sessions, Advanced = 982 sessions, Expert = 1,741 sessions. All instructor experience groups showed comparable mean mood improvements with overlapping distributions. Instructor experience was not a statistically significant predictor of post-class mood in the multilevel model (M5, Section 3.2). This figure is included as supplementary material because the variable did not improve model fit and was not retained in the final model. [file Image_1.jpeg]
